# Regulated necrosis role in inflammation and repair in acute kidney injury

**DOI:** 10.3389/fimmu.2023.1324996

**Published:** 2023-11-24

**Authors:** Juan Guerrero-Mauvecin, Natalia Villar-Gómez, Sandra Rayego-Mateos, Adrian M. Ramos, Marta Ruiz-Ortega, Alberto Ortiz, Ana B. Sanz

**Affiliations:** ^1^ Laboratorio de Nefrología Experimental, Instituto de Investigación Sanitaria-Fundación Jimenez Diaz (IIS-FJD), Universidad Autonoma de Madrid, Madrid, Spain; ^2^ Redes de Investigación Cooperativa Orientadas a Resultados en Salud (RICORS2040), Madrid, Spain; ^3^ Cellular Biology in Renal Diseases Laboratory, IIS-FJD-Universidad Autónoma, Madrid, Spain; ^4^ Department of Medicine, Universidad Autonoma de Madrid, Madrid, Spain; ^5^ Instituto Reina Sofia en Investigación en Nefrología (IRSIN), Madrid, Spain

**Keywords:** acute kidney injury, chronic kidney disease, cell death, fibrosis, inflammation, tissue repair

## Abstract

Acute kidney injury (AKI) frequently occurs in patients with chronic kidney disease (CKD) and in turn, may cause or accelerate CKD. Therapeutic options in AKI are limited and mostly relate to replacement of kidney function until the kidneys recover spontaneously. Furthermore, there is no treatment that prevents the AKI-to-CKD transition. Regulated necrosis has recently emerged as key player in kidney injury. Specifically, there is functional evidence for a role of necroptosis, ferroptosis or pyroptosis in AKI and the AKI-to-CKD progression. Regulated necrosis may be proinflammatory and immunogenic, triggering subsequent waves of regulated necrosis. In a paradigmatic murine nephrotoxic AKI model, a first wave of ferroptosis was followed by recruitment of inflammatory cytokines such as TWEAK that, in turn, triggered a secondary wave of necroptosis which led to persistent kidney injury and decreased kidney function. A correct understanding of the specific forms of regulated necrosis, their timing and intracellular molecular pathways may help design novel therapeutic strategies to prevent or treat AKI at different stages of the condition, thus improving patient survival and the AKI-to-CKD transition. We now review key regulated necrosis pathways and their role in AKI and the AKI-to-CKD transition both at the time of the initial insult and during the repair phase following AKI.

## 1 Overview of AKI

Acute kidney injury (AKI) is defined by a rapid decline of renal function, resulting in increased serum creatinine levels or decreased urine output below certain thresholds (1, 2). AKI may be triggered by pre-renal, renal, and post-renal (urinary tract obstruction) causes. Renal causes like drugs, sepsis/shock and ischemia-reperfusion injury (IRI) that may lead to tubular cell death include from direct tubular toxicity to crystal-induced kidney injury (2, 3). Even though the loss of renal function is at least partially reversible in most patients who survive, the mortality rate of AKI remains high (over 50%) (4, 5). Moreover, AKI episodes favor the progression to chronic kidney disease (CKD), and CKD is a risk factor for AKI (6, 7). Cell death and inflammation play a key role in AKI. Systemic and local inflammation can cause tubular cell death and AKI, and dying tubular cells may trigger a secondary inflammatory response that may further amplify tubular cell death (8). Several pathways of regulated necrosis, such as necroptosis, ferroptosis and pyroptosis, have emerged as proinflammatory cell death pathways since dying cells release proinflammatory factors that amplify tissue injury (8, 9). A role for regulated necrosis has been observed in preclinical AKI induced by sepsis, IRI and nephrotoxicity, and there is evidence of its occurrence in humans (10–12). The desired outcome of an AKI episode is complete recovery of kidney structure and function, but in most cases, this does not occur. Tubular cells with sublethal damage can either completely recover their function and phenotype or, if regeneration is defective, they may evolve to a profibrotic phenotype, contributing to CKD progression (13). Now, we review the role of regulated necrosis pathways in inflammation and repair in AKI.

## 2 Adaptive and maladaptive repair after AKI: AKI and CKD progression

The AKI-to-CKD transition may be related to factors that depend on the nature and intensity of the stimulus or its interactions with the kidney tissue and the specific cellular niches affected. Mechanistically, all kinds of AKI centrally impact the tubular epithelium by producing cell stress and death. Thus, tubular cells are the epicenter from which damage expands to other areas of the kidney (14). Adaptive tissue repair after an AKI episode depends on an orderly balance between tubular cell death and the proliferation of survival cells that should maintain a healthy tubular cell phenotype. The persistence or recurrence of damaging stimuli shifts this balance towards increased cellular stress and a state of maladaptive repair characterized by ongoing cell death, persistent inflammation, and tissue fibrosis and aging, all of which contribute to chronicity. Extensive kidney cell death without recovery depletes the normal functional epithelia which is replaced by scar tissue and fibrosis. Therefore, consistent with the central role of cell death in promoting AKI initiation and the AKI-to-CKD transition, cell death is a primary therapeutic target to improve AKI outcomes.

## 3 Regulated necrosis pathways

Necroptosis, ferroptosis and pyroptosis are the main regulated necrosis pathways and they are interrelated.

### 3.1 Necroptosis

Necroptosis results in cell swelling, and membrane disruption and triggers an inflammatory response (15). The best characterized trigger is activation of TNF superfamily receptors by their ligands, recruiting TRADD, TRAF2 and RIPK1, among other proteins. In a pro-survival context, cellular inhibitors of apoptosis proteins (CIAPs) and the Linear Ubiquitin Chain Assembly Complex (LUBAC) drive the polyubiquitination of RIPK1, activating the NF-кB pathway, which promotes cell proliferation and survival (16, 17). On the contrary, when NF-kB activation is inhibited, RIPK1 is deubiquitinated and associates with FADD and procaspase-8, resulting in cleavage of procaspase-8, generating active caspase-8 that results in execution of apoptosis (18, 19). In the absence of caspase-8 activity, RIPK1 is not cleaved, and associates with RIPK3, resulting in transphosphorylation or autophosphorylation of RIPK3, and the formation of the necrosome, which phosphorylates MLKL, resulting in MLKL oligomerization and translocation to the plasma membrane (20). Membrane-bound pMLKL oligomers form pores that lead to calcium influx and the release of damage-associated patterns (DAMPs), that trigger an inflammatory response (21–24).

### 3.2 Ferroptosis

Ferroptosis is an iron-dependent regulated form of necrotic cell death characterized by excessive lipid peroxidation of organelle and cell membranes that causes their disruption, leading to cell death and the release of DAMPs that trigger an inflammatory response (15). Unlike other regulated forms of cell death, ferroptosis is not regulated by specific molecular mediators, but it depends on the redox balance and the cellular antioxidant defense. Key factors involved in triggering ferroptosis, include low levels of the antioxidant molecule glutathione (GSH), impaired GPX4 activity, imbalanced polyunsaturated fatty acid (PUFA) contents and iron (Fe) availability (25). Inhibition of the Xc- antiporter can trigger ferroptosis, since it allows cystine import into cells, which is indispensable for synthesis of GSH, a GPX4 cofactor (26). GPX4 activity is the main cellular antioxidant defense against ferroptosis and reduces lipid hydroperoxides (L-OOH) on their corresponding lipid alcohols (L-OH) (27, 28). Kidney tubular cells are especially dependent on GPX4 and acquired GPX4 deficiency triggers AKI (27). Other mediators of ferroptosis include PUFAs that can be esterified into membrane phospholipid (PL) and become oxidative susceptible species (PL-PUFAs). The activation and esterification of PUFAs are mediated by acyl-CoA synthetase long-chain family member 4 (ACSL4) and lysophosphatidylcholine acyltransferase 3 (LPCAT3) (29–31). An increased content of these species could increase the susceptibility to ferroptosis since PL-PUFAs can be oxidized by lipoxygenases (LOX) forming lipid peroxides (32, 33). Another key regulator of ferroptosis is iron, which is necessary for LOX activity and can trigger Fenton reactions creating more lipid peroxides (34, 35). Ferroptosis may be propagated to adjacent tubular cells as a synchronized wave of cell death, where a single tubular cell may trigger teh death to the whole tubule (36). This synchronized wave of cell death seems to be propagated through calcium signals and might be stimulus-dependent, was observed in erastin-induced GSH depletion but not during GPX4 inhibition (37). Understanding the mechanisms that mediate the spread of ferroptosis through cell populations will help us to identify new ferroptosis inhibitors.

### 3.3 Pyroptosis

Pyroptosis is characterized by membrane rupture and pro-inflammatory effects. Gasdermins (GSDM) are the main effectors of pyroptosis. Activated GSDM inserts into cell membranes and forms pores leading to the release of cytokines, alarmins and DAMPs, cell membrane rupture and cell death (12). GSDM activation can be mediated by canonical and non-canonical pathways. The canonical pathway involves the activation of Toll-like receptors (TLRs), which induce the expression of inflammasome components and pro-inflammatory cytokines. In parallel, inflammasome sensors such as NLRP3 are activated by a variety of DAMPs and PAMPs and recruit adaptor proteins, CARD and pro-caspase-1, to form the inflammasome complex and activate caspase-1. Caspase 1 cleaves pro-IL-β, pro-IL-18, and GSDMD promoting membrane pore formation, cytokine release and lytic cell death (12, 38). In the non-canonical pathway, caspases-4, -5 and -11 are directly activated by intracellular lipopolysaccharide, independent of the inflammasome, and they consequently cleave GSDMD to execute pyroptosis, without IL-1β and IL-18 cleavage (39). Pyroptosis can interact with apoptosis since caspase-3 and -7 may activate GSDME and caspase-8 GSDMD (40, 41). Pyroptosis is associated with host antimicrobial defense, but it can also be involved in sterile diseases such as atherosclerosis and neurodegenerative diseases (42–44).

### 3.4 Molecular interactions between different forms of regulated necrosis and apoptosis

As recently reviewed, there are multiple interactions between different modalities of regulated necrosis and apoptosis, beyond the fact that regulated necrosis may recruit inflammation-driven cell death, that should be accounted for when designing therapeutic interventions (8). Examples include the several roles of caspases in cell death modalities ranging from apoptosis to necroptosis or pyroptosis, final common pathways such as the recruitment of NINJ1 to lyse the cell membrane and common protective mechanisms such as preservation of cell membrane integrity by ESCRT-III (8).

## 4 Regulated necrosis pathways and induction of AKI

Contrary to the old-fashioned assumption that apoptosis accounts for the majority of dying cells in AKI, much evidence has emerged in the last decade for a predominant role and contribution of regulated forms of necrotic cell death, in particular necroptosis and ferroptosis. Therefore, elucidating their relative contribution and interconnection in AKI will allow for the development of more precision targeted therapies. To this end, several studies have investigated the involvement of regulated necrosis pathways in experimental AKI through interference with the activity of key components of the molecular pathway.

In general, the role of ferroptosis and necroptosis in different models of AKI has been clearly demonstrated. During folic acid-induced nephrotoxic AKI (FA-AKI), a first wave cell death by ferroptosis induces an inflammatory response that triggers a secondary wave of cell death by necroptosis in which the inflammatory cytokine TWEAK activation of the Fn14 receptor is involved (45, 46) In rhabdomyolysis-induced kidney injury, ferroptosis appears to be the dominant pathway, as ferrostatin-1 improved renal function while the necroptosis inhibitor necrostatin-1 had no effect (47, 48). This makes sense, since myoglobin is a heme-containing protein, i.e., a source of excess iron. In both IRI-AKI and crystal nephropathy, targeting necroptosis, ferroptosis and mitochondrial permeability transition pore-regulated necrosis (MPTP-RN) were protective (36, 49–53). Moreover, necroptosis and ferroptosis may be interconnected in IRI-AKI, since MLKL-deficient mice subjected to renal IRI showed an earlier upregulation of ACSL4, a potential mediator of ferroptosis (54), supporting the notion that combined therapy may be more effective than targeting a single pathway. In murine cisplatin-AKI, deficiency of RIPK3 or MLKL resulted in improved renal function, pointing out necroptosis as a major mechanism of tubular cell death (55), but there may also be a link with other regulated necrotic pathways, as some studies suggest a role for ferroptosis (56–58). In sepsis-AKI, RIPK3 aggravated kidney injury in a MLKL-independent manner by promoting mitochondrial dysfunction via NOX4 upregulation, but the contribution of tubular cell death was not clearly demonstrated (59).

By contrast, the role of pyroptosis in AKI is controversial. Some studies have found that caspase-11 expression and cleavage of GSDMD or GSDME were increased in both IRI-AKI and cisplatin-AKI. Caspase-11-, GSDMD- or GSDME-deficient mice were protected from cisplatin-AKI, and specifically GSDME deficiency also ameliorated injury in IRI-AKI (60–62). In contrast, an independent group reported that in both GSDMD- and GSDME-deficient mice the severity of IRI-AKI, cisplatin-AKI and calcium oxalate-AKI were increased due to activation of necroptosis (63). In addition, whether tubular cells express pyroptosis proteins is disputed (60, 61, 63, 64). Further studies should clarify which cells activate pyroptosis in AKI, whether targeting pyroptosis is truly protective and which is the optimal way to target pyroptosis. Multicenter preclinical trials may help address these discrepancies (65).

Moreover, further research should address the *in vivo* relationships between different modes of regulated necrosis in different forms of AKI: which forms of cell death occur initially and how do they trigger similar or another form of cell death in neighboring cells (8), what is the impact of the nature and strength of the stimulus, along with the presence of co-stimulatory factors, on these dynamics (8), and above all, how these preclinical observations relate to the clinical situation, in which the time-course of injury is frequently unclear and different insults may pile up in the same patient at different time points following the initial injury. Clinical translation sorely needs soluble biomarkers of different modalities of regulated necrosis that allow a dynamic follow-up of ongoing types of regulated necrosis and their response to different therapeutic interventions.

## 5 Interaction between regulated necrosis and inflammation in AKI

During homeostatic and developmental scenarios, apoptosis-driven removal of damaged or unneeded cells regulates cell populations in the kidney and other organs. This process involves efferocytosis, a non-inflammatory mechanism where cell surface molecules (eat-me signals) are recognized by macrophages that engulf and clear apoptotic cells (66). In contrast, regulated necrosis pathways are characterized by the lack of early engulfment, formation of pores in cellular membranes, membrane lysis and the consequent release of DAMPs, which engage an inflammatory response that amplifies injury, in a process termed necroinflammation (67). DAMPs released during regulated necrosis may also be immunogenic and are thought to play a role in autoimmune diseases such as lupus nephritis. Ferroptosis has the most proinflammatory and immunogenic potential, since it both releases DAMPs and lipid peroxides but also propagates cell death in a synchronized manner (37), whereas necroptosis generates both pro-inflammatory cytokines such as IL-1β (68) and anti-inflammatory cytokines such as IL-33 and CXCL1 (68–70).

During AKI, damaged and dying kidney parenchymal cells release DAMPs, which can activate pattern recognition receptors such as TLRs or NOD-like receptors proteins (NLRPs) on kidney resident immune cells like dendritic cells and macrophages, as well as chemokines and cytokines, thus attracting and activating leucocytes and amplifying the inflammation (71). The resulting inflammation depends on the nature and the persistence of the stimulus, as well as the renal compartment that is affected (72). Likewise, the specific secondary mediators differ among different forms of regulated cell death, which contributes to the complexity and heterogeneity of their impact on kidney injury (73). For example, in FA-AKI, ferroptosis is activated at early time points, and in addition to inducing cell death, it also triggers the expression of proinflammatory mediators such as Fn14 (TWEAK receptor), which promote a second wave of cell death by necroptosis (45). Similarly, in both cisplatin- and IRI-AKI, MLKL and RIPK3 deficiency reduces necroptosis, and also the tubular expression of inflammatory cytokines, such as TNFα, that trigger necroptosis (55, 74). In this regard, a novel inhibitor of RIPK1, Cpd-71, prevented cell death and inflammation in cisplatin-AKI (75).

Conversely, inflammation can trigger cell death, as characterized for IRI-AKI, where prostaglandin activation of the E-prostanoid 3 receptor (EP3) in myeloid cells promotes the release of inflammatory cytokines that activate necroptosis and necroinflammation in tubular cells (76). Some stimuli activate both inflammation and cell death, as illustrated by cisplatin- and IRI-AKI, where the interaction of the gastrin-releasing peptide receptor with TLR4 in tubular cells activates STAT1 to promote the expression of MLKL and CCL2, leading to necroptosis and inflammation (77).

On the other hand, RIPK3 can promote kidney inflammation independently of necroptosis. In FA-AKI, RIPK3 deficiency reduced inflammation but not cell death at early time points when necroptosis had not yet been recruited as a key cell death pathway (78). RIPK3 also mediated kidney inflammation after systemic injection of TWEAK, a model of inflammation that does not cause kidney cell death or dysfunction (78). Additionally, in experimental sepsis, where inflammation plays a key role, RIPK3, but not MLKL. mediated kidney injury and dysfunction (59, 79).

Overall, during AKI, cell death and inflammation are interconnected pathways, and an in-depth knowledge of this connection is necessary to optimize therapeutic approaches to AKI.

## 6 Regulated necrosis and tissue repair after AKI

**Figure 1:**
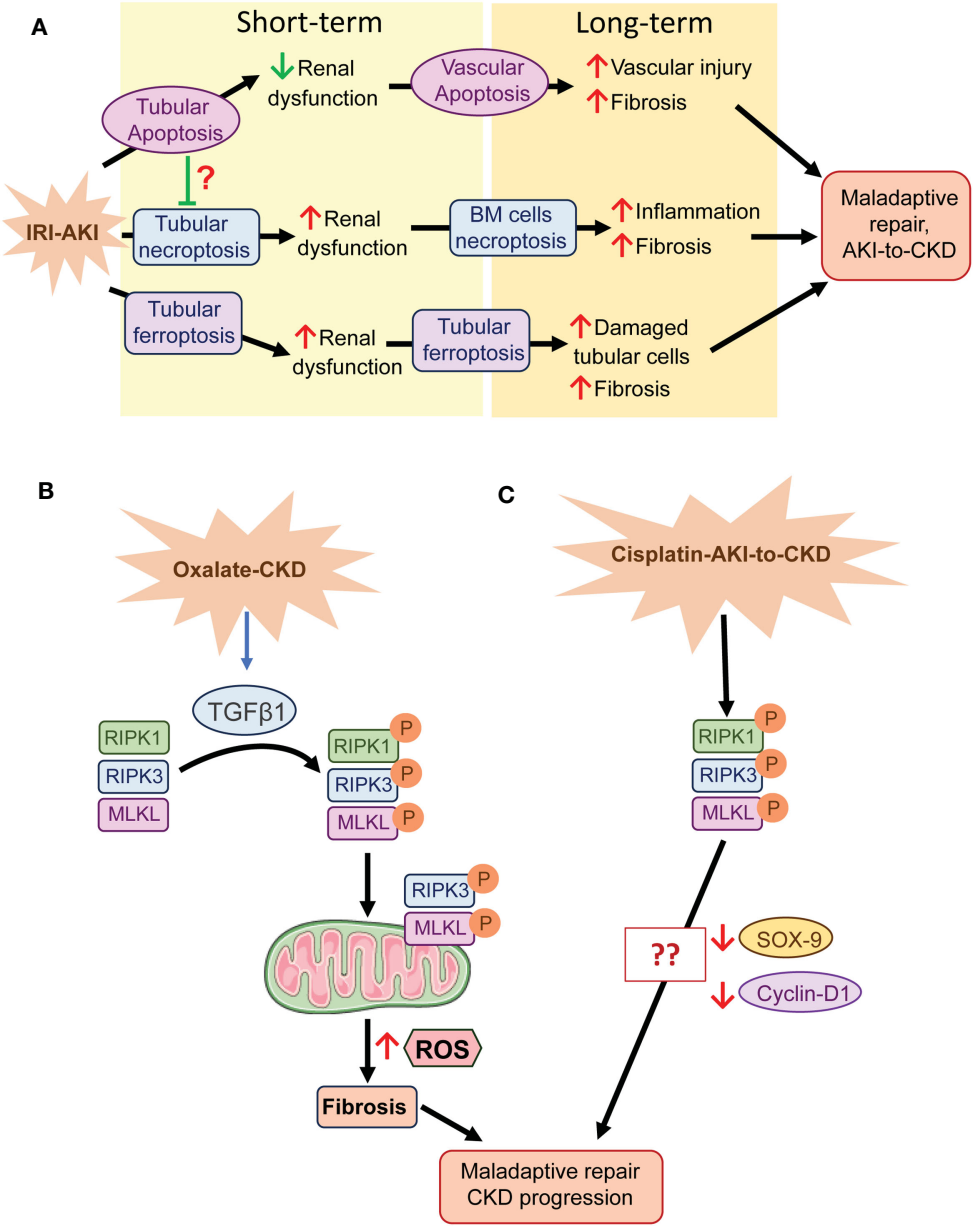
Regulated necrosis and tissue repair after AKI. **(A)** In IRI-AKI, several cell death pathways are activated. Apoptosis may play a beneficial role in the short term, probably regulating cellular homeostasis, but in the long term it favors fibrosis through cell death of perivascular cells. However, tubular necroptosis and ferroptosis play a detrimental role in both the short and long term, probably mediated by the necroinflammatory loop. **(B)** In oxalate CKD, the necrosome is activated by TGFβ1 and RIPK3 and MLKL translocate to the mitochondrial membrane where they promote ROS generation, fibrosis and consequent maladaptive repair and CKD progression. **(C)** The necrosome is activated in cisplatin-induced AKI-to-CKD transition, but its specific role in CKD progression is unknown, although, SOX-9 and cyclin-D1 downregulation may be involved. BM, Bone marrow.

Apoptosis of tubular cells was widely investigated in the 20th and early 21^st^ centuries as a mechanism of regulated cell death driving AKI and the AKI-to-CKD. However, none of the therapeutic approaches made it to the clinic and apoptosis may also lead to loss of other cell types or clear excess tubular cells generated by cell proliferation following injury as well as irreversibly damaged cells (80). Thus, whereas caspase-3 deficiency increased the severity of early IRI-AKI, probably by shifting cell death to necrosis, it reduced long-term renal damage by inhibiting endothelial apoptosis, vascular rarefaction, and fibrosis (81) (**Figure 1A**).

In contrast, regulated necrosis, which is always considered pathological, may offer new therapeutic alternatives to treat AKI or the AKI-to-CKD transition (8). Several preclinical studies have associated regulated cell death with maladaptive repair following AKI (82). In IRI-AKI inhibition of the necroptotic pathway improved kidney function early in IRI-AKI and the progression to fibrosis in the long term, where bone marrow cells play a key role, suggesting that the inflammation associated to regulated necrosis favors the AKI-to-CKD transition (74). Single-cell transcriptomics showed that tubular cells undergoing maladaptive repair after prolonged ischemia presented an enrichment of ferroptosis and pyroptosis pathways, supporting their role in maladaptive repair (83). Another report, which combined a single-cell transcriptomic study with genetic approaches, demonstrated that chronic inflammation after IRI-AKI downregulated the gene expression of glutathione metabolism components triggering ferroptotic stress and identified GPX4 as a key coordinator of kidney repair and regeneration (84) (**Figure 1A**). Deficiency of IL-18, a component of NLRP3-inflammatory response, ameliorated the early phase of necroptosis as well as later tissue regeneration in murine FA-AKI (85).

In CKD patients and mice with oxalate-induced CKD increased renal levels of RIPK1, RIPK3, MLKL correlated with increased extracellular matrix (ECM) production and declining kidney function (86). Both pharmacological and genetic inhibition of RIPK3 diminished ECM accumulation in oxalate-induced CKD, adenine diet-induced renal fibrosis and, unilateral ureteral obstruction (86, 87), showing the involvement of necroptosis in kidney fibrosis. Moreover, profibrotic factors recruit RIPK3 and MLKL to mitochondria resulting in mitochondrial dysfunction and reactive oxygen species (ROS) production in murine fibroblasts stimulated with TGFβ1 and in oxalate-induced CKD (86) (**Figure 1B**).

Additionally, necroptosis was identified as a determinant pathway in the progression of CKD in cisplatin-AKI, although the specific mechanisms of necroptosis in this model were not described (88). Activation of specific transcriptional repair programs, including STAT3 and SOX-9 pathways, contribute to cell repair during regeneration following AKI (89–91). In murine cisplatin-AKI, 7-Hydroxycoumarin diminished renal necroptosis by modulating the RIPK1/RIPK3/MLKL pathway, and increased tissue repair through upregulation of cyclin D1 (92). Moreover, in cultured HK2 cells, SOX-9 deficiency reduced the beneficial effect of 7-Hydroxycoumarin against cisplatin cytotoxicity (92), suggesting a potential relationship between SOX-9, necroptosis, and tissue repair (**Figure 1C**).

Following AKI, damaged tubular cells may become senescent and display the secretory associated senescence phenotype (SASP), characterized by profibrotic and proinflammatory factors, which spread cellular senescence to neighboring cells and contribute to the propagation of kidney damage (13). Cellular communication network factor 2 (CCN2) is a component of the SASP that induces senescence in cultured tubular cells, is involved in experimental renal fibrosis following IRI- and FA-AKI and can activate the RIKP3/NLRP3 pathway in the acute phase of FA-AKI (93–95). In aging mice, FA-AKI is more severe compared to young mice, and this has been linked to cellular senescence related mechanisms, including increased expression of SASP components such as CCN2, and to upregulation of necroptosis cell-death pathways (96), suggesting a link between senescence and necroinflammation in renal injury. In this line, deficiency of IL-18, a component of NLRP3-inflammatory response, ameliorated the early phase of necroptosis as well as later tissue regeneration in murine FA-AKI (85).

The therapeutic effect of extracellular vesicles (EVs) on renal recovery after AKI has also been linked to modulation of regulated necrosis pathways (97–99). Exosomes from human umbilical cord-derived mesenchymal stem cells modulated necroptosis through miR-874-3p attenuating HK2 cell injury and enhancing repair following cisplatin stimulation (100). In murine LPS-induced AKI, treatment with adipose-derived EVs reduced renal inflammation and pyroptosis and promoted tubular cell repair through miR-21-5p/TLR4, blocking the NF-κB/NLRP3 pathway (101).

Overall, these results support a key role of regulated cell death in the regeneration and repair phase of AKI.

## 7 Conclusion

**Figure 2:**
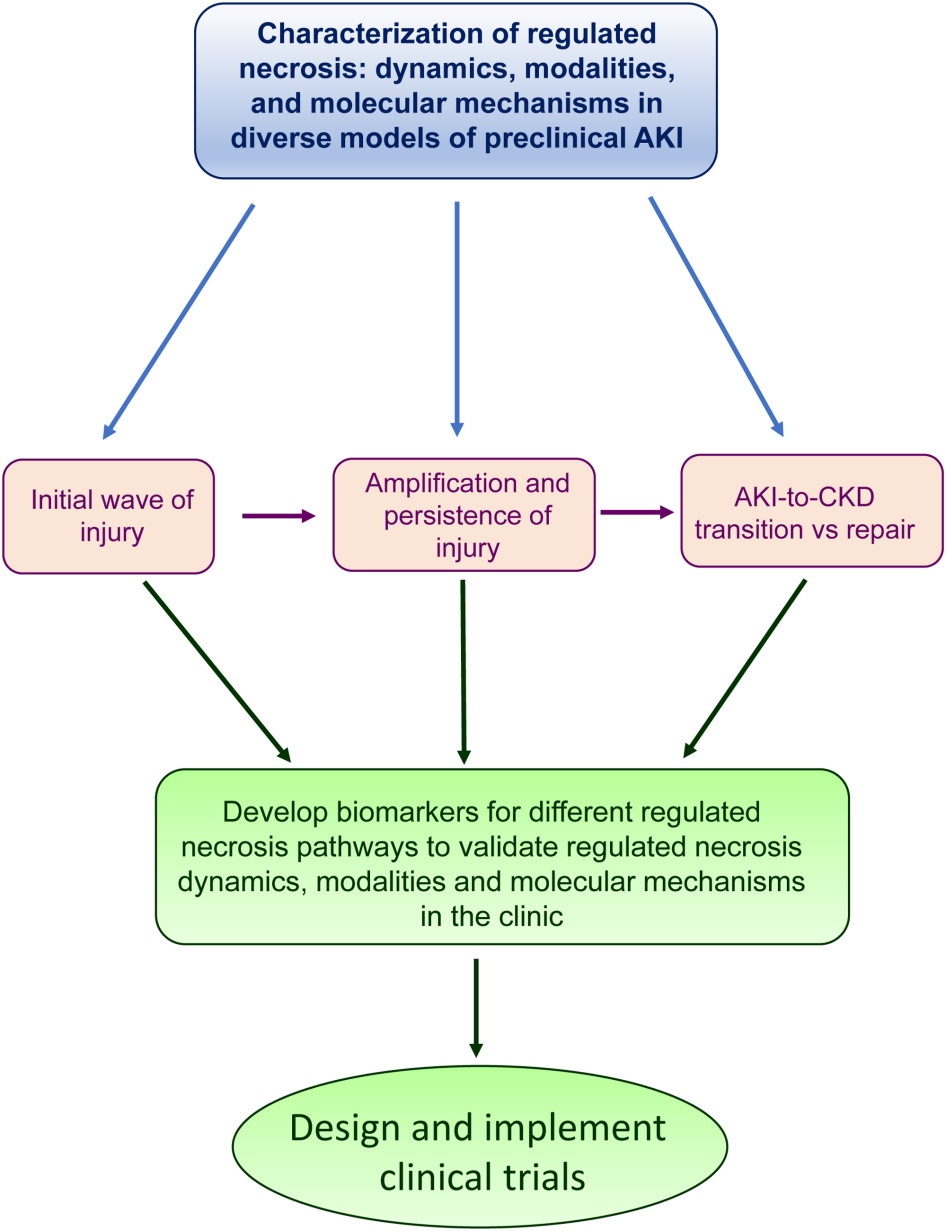
The path to clinical translation of findings in preclinical models. The findings described in preclinical models about the role of regulated necrosis in the different phases of AKI could help to develop novel biomarkers in the clinic.

In conclusion, there is accumulating evidence for a role of different modalities of regulated necrosis in the initial phase of AKI, the amplification and persistence of injury following the initial insults, the repair phase from AKI and the AKI-to-CKD transition. A better understanding of the molecular mechanisms involved in different phases of AKI following diverse insults may identify novel therapeutic targets. However, clinical development should rely on the development of biomarkers that allow to monitor the activation of different modalities of regulated necrosis as well as their response to therapeutic interventions in humans (**Figure 2**).

## Author contributions

JG: Writing – original draft, Writing – review & editing. NV: Writing – original draft, Writing – review & editing. SR: Writing – original draft, Writing – review & editing. AR: Writing – original draft, Writing – review & editing. MR: Writing – original draft, Writing – review & editing. AO: Conceptualization, Supervision, Writing – original draft, Writing – review & editing. AS: Conceptualization, Funding acquisition, Supervision, Writing – original draft, Writing – review & editing.
